# Transgenerational effects of UV-B radiation on egg size, fertilization, hatching and larval size of sea urchins *Strongylocentrotus intermedius*

**DOI:** 10.7717/peerj.7598

**Published:** 2019-08-26

**Authors:** Jingyun Ding, Lingling Zhang, Jiangnan Sun, Dongtao Shi, Xiaomei Chi, Mingfang Yang, Yaqing Chang, Chong Zhao

**Affiliations:** Key Laboratory of Mariculture & Stock Enhancement in North China’s Sea, Ministry of Agriculture and Rural Affairs, Dalian Ocean University, Dalian, China

**Keywords:** UV-B, Transgenerational effect, *Strongylocentrotus intermedius*, Adaptation

## Abstract

Transgenerational effects are important for phenotypic plasticity and adaptation of marine invertebrates in the changing ocean. Ultraviolet-B (UV-B) radiation is an increasing threat to marine invertebrates. For the first time, we reported positive and negative transgenerational effects of UV-B radiation on egg size, fertilization, hatchability and larval size of a marine invertebrate. *Strongylocentrotus intermedius* exposed to UV-B radiation showed positive transgenerational effects and adaptation on egg size, hatching rate and post-oral arm length of larvae. Negative transgenerational effects were found in body length, stomach length and stomach width of larvae whose parents were exposed to UV-B radiation. Sires probably play important roles in transgenerational effects of UV-B. The present study provides valuable information into transgenerational effects of UV-B radiation on fitness related traits of sea urchins (at least *Strongylocentrotus intermedius*).

## Introduction

Marine invertebrates have complex life cycles alternating between the short-lived embryonic and larval stages and the long-lived adult stage. The performance in a life history stage can lead to significant positive or negative carryover effects on subsequent life history stages, also known as the developmental domino phenomena (Byrne, 2012). Carryover effects can arise within a generation, for example, embryonic and larval experiences can affect the success of juveniles and adults; as well as across a generation (transgenerational effects), with phenotypic changes in offspring in response to the environmental stress experienced by one or both parents (Kovalchuk, 2012; Shama & Wegner, 2014; Munday, 2014). Transgenerational effects of a few environmental factors have been investigated in marine invertebrates, including salinity (Davis, 1958), heavy metals (Untersee & Pechenik, 2007), and temperature (Byrne, 2011).

To our knowledge, however, it remains unknown on whether transgenerational effects of solar ultraviolet-B radiation (UV-B, 280–315 nm) radiation exist in marine invertebrates, in spite of its ecological importance. UV-B becomes an increasing threat to marine invertebrates, because of the anthropogenic gases induced ozone depletion (Day & Neale, 2002; Manney et al., 2011). At least 10% UV-B can penetrate to seawater to a depth of 16 m (Tedetti & Sempéré, 2006), highlighting the possible impacts to marine invertebrates in intertidal and shallow water. Most studies focused on real-time effects of UV-B of marine organisms (Lamare, Burritt & Lister, 2011). UV-B radiation probably brings about long-term and transgenerational carryover effects on the fitness of marine invertebrates (Zhao et al., 2018a), because UV-B causes cellular damage by oxidizing proteins, DNA and membrane lipids cannot immediately recover or even irreversible (Adams & Shick, 2001).

Sea urchins are a group of ecologically important marine invertebrates in structuring marine benthic communities, both as grazers and prey (Pearse, 2006). Species inhabit intertidal and shallow waters are vulnerable to UV-B radiation, although primary avoidance and protection strategies provide them with the ultimate safeguard against UV-B (Lamare, Burritt & Lister, 2011), including behavioral responses (e.g., covering behavior) (Sigg, Lloyd-Knight & Boal, 2007), photoprotectants (e.g., mycosporine-like amino acids (MAAs)), DNA repair mechanisms (e.g., photoreactivation). DNA dimers (Cyclobutane pyrimidine dimers and 6–4 photoproducts) formation is a key DNA damage to UVR exposure in eggs, embryos and larvae of sea urchins (Ravanat, Douki & Cadet, 2001; Lamare, Burritt & Lister, 2011). This probably results in fertilization interference, delayed development (Lamare, Burritt & Lister, 2011), impaired skeletal formation (Bonaventura et al., 2005; Nahon et al., 2009) and apoptosis (Lesser, Kruse & Barry, 2003). The sea urchin *Strongylocentrotus intermedius*, a representative keystone ecosystem engineer in intertidal and shallow seas around Japan, Korea, northeastern China and Far East Russia (Agatsuma, 2013), are sensitive and susceptible to UV-B radiation (Zhao et al., 2018a) through their entire biological development, including gametes, fertilization and embryonic and larval development (Lamare, Burritt & Lister, 2011). It is especially essential to consider of the annual dose of UV-B radiation around the areas, where *Strongylocentrotus intermedius* exist. This was ~6 MJ/(m^2^ ∙ yr) (~18.9 μW ∙ cm^−2^) at Tokyo (Bais et al., 2015). Although the data of UV-B radiation was observed by land-based spectroradiometers. Recently, short-term (1 h) UV-B radiation (20 μW ∙ cm^−2^) showed significantly adverse effects on survival, food consumption, test diameter, test height, test height:test diameter, gonad weight and crude protein of gonads of *Strongylocentrotus intermedius*, despite the absence of UV-B radiation for 8 weeks (Zhao et al., 2018a). Therefore, we hypothesized that transgenerational effects of short-term (1 h) UV-B radiation (20 μW ∙ cm^−2^) exist in *Strongylocentrotus intermedius* (for example, egg size, fertilization, hatchability and larval size). The main aim of the present study is to test whether transgenerational effects short-term (1 h) UV-B radiation (20 μW ∙ cm^−2^) exist in sea urchins (at least *Strongylocentrotus intermedius*). We asked that (1) whether UV-B radiations have significant transgenerational effects on egg size, fertilization, hatching and larval size of *Strongylocentrotus intermedius*, (2) whether the transgenerational effects are influenced by sires.

## Methods

### Sea urchins

The present study was an extension of our previous study (Zhao et al., 2018a). The source of sea urchins and experimental design were fully described in Zhao et al. (2018a) and briefly described as follows:

*Strongylocentrotus intermedius* (test diameter = 44.97 ± 1.23 mm) were transported from the hatchery of Dalian Haibao Fishery Company to the Key Laboratory of Mariculture and Stock Enhancement in North China’s Sea, Ministry of Agriculture and Rural Affairs at Dalian Ocean University. The sea urchins were cultured in a tank (length × width × height: 180 × 100 × 80 cm) with aerated seawater until the experiment started. They were fed wild fresh *Saccharina japonica* and *Ulva lactuca* ad libitum under natural photoperiod (from 12 h light:12 h dark). The seawater was changed every 3 days.

### Experimental design

Sea urchins were exposed to UV-B irradiance levels at zero and 20 μW ∙ cm^−2^ for 1 h using a UV-B lamp (280–315 nm, TL 40W/12 RS; Philips Co., Hamburg, Germany) (Shi et al., 2018). The intensity of UV-B radiation (20 μW ∙ cm^−2^) were set by regulating the distance between UV-B lamp and the surface of the water *(*Adams, 2001).The control group was set as zero μW ∙ cm^−2^. The UV-B exposures were carried out in an isolated box with no environmental UV-B radiation involved. *Strongylocentrotus intermedius* were placed into randomly distributed cages (length × width × height: 75 × 43 × 43 cm) in a tank (one ton in volume) after each UV-B radiation (zero and 20 μW ∙ cm^−2^). All sea urchins were cultured in aerated seawater with almost no UV-B radiation from solar radiation (zero μW ∙ cm^−2^) in a room for 10 months until the breeding experiment started, only except for several days with very little UV-B radiation (0–0.5 μW ∙ cm^−2^) (Shi et al., 2018). They were fed wild fresh *Saccharina japonica* and *U. lactuca* ad libitum under natural photoperiod (from 12 h light:12 h dark) at the laboratory during the 10 months after the UV-B radiation (zero and 20 μW ∙ cm^−2^). The seawater was changed every 3 days.

Breeding experiments were carried out on June 9, 2017. Three mating groups were set as follows: group A (♀0 × ♂0 μW · cm^−2^), group B (♀20 × ♂20 μW · cm^−2^) and group C (♀20 × ♂0 μW · cm^−2^). Three families were produced for each mating group (*N* = 3).

Spawning, fertilization, hatching and larval culture were followed our previous study (Zhao et al., 2018b), which were briefly described as follows:

Spawning was induced by the injection of one mL KCl (0.5M) into the coelom via the peristomial membrane. Eggs were collected in filtered seawater and sperm were collected dry. Spawning time was limited to 30 min to ensure gamete quality. The same amount of eggs was collected in each bottle. Collected eggs were filtered and transferred to 3 L bottles at the appropriate temperatures using a fine silk net (mesh size: 106 μm). According to the mating design, 500 mL of egg suspension (~0.1 million eggs) were collected from the well mixed seawater in the 3 L bottle and fully mixed with ~50 μL dry sperm for fertilization. According to the method of Suckling et al. (2015), our preliminary experiment indicated that this egg: sperm ratio is optimal for the fertilization of *Strongylocentrotus intermedius*. The egg:sperm ratio was kept approximately the same across the experiment, although sperm concentration was not calculated. The breeding experiments described above were exactly repeated using different dams and sires three times in each mating group.

Fertilized eggs were transferred into nine separated cylindrical cages (226 cm^2^ × 8 cm, ~5 L). The embryo density was ~20 ind · mL^−1^. After measurement of fertilization and hatchability, we collected hatched blastulae in the upper 3/4 of the seawater with dead and unhatched blastulae removed. Larvae were cultured in nine containers of ~10 L (bottom diameter: 23 cm, height: 24 cm) with the density of ~0.5 ind · mL^−1^. Larvae in all tanks were fed with the microalga *Chaetoceros gracilis* three times a day in the weakly aerated seawater.

### Egg diameter

Egg diameter was measured before fertilization using a microscope (DS-Ri1; Canon, Tokyo, Japan). A total of 30 eggs (10 eggs for each family) were measured for mating groups A (♀0 × ♂0 μW · cm^−2^), B (♀20 × ♂20 μW · cm^−2^) and C (♀20 × ♂0 μW · cm^−2^), respectively.

### Fertilization and hatching rates

Fertilization and hatching rates were measured 4 and 27 h after fertilization using a microscope (DS-Ri1; Canon, Tokyo, Japan). The methods of measurement and calculation were according to our previous study (Zhao et al., 2018b), which are summarized as follows:

$$F\left( \% \right)\, = \,{x \over y}\, \times \,100$$

where *F* = fertilization rate, *x* = number of embryos in cleavage and *y* = total number of embryos and eggs.

$$H\left( \% \right)\, = \,{x \over y}\, \times \,100$$

where *H* = hatching rate, *x* = number of prism larvae and *y* = total number of larvae and embryos.

### Larval size

**Figure 1:**
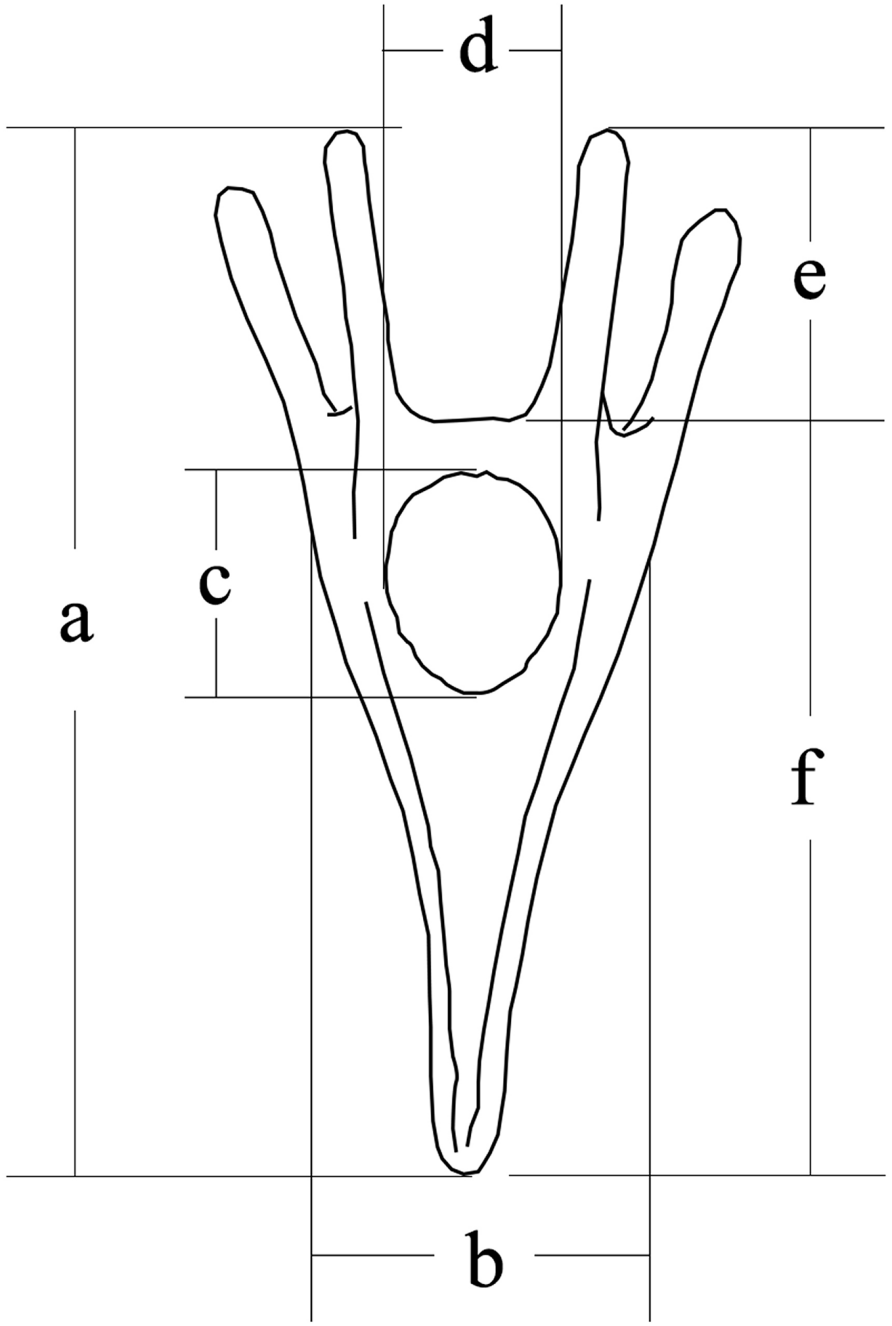
Conceptual diagram of larval size measurements. Letters a, b, c, d, e and f refer to larval length, larval width, stomach length, stomach width, postoral arm length and body rod length, respectively.

Larval size was measured 5 days after fertilization, according to our previous study (Zhao et al., 2018b). We measured larval length, larval width, stomach length, stomach width, post-oral arm length and body rod length of sea urchins (50 larvae for each family) using a microscope (DS-Ri1; Canon, Tokyo, Japan) (Fig. 1).

### Statistical analysis

All data were tested for normal distribution and homogeneity of variance before statistical analysis. Egg size, larval length, stomach length, body rod length and post-oral arm length were analyzed using nested univariate ANOVA. LSD’s multiple comparisons were carried out when significant difference was found in nested univariate ANOVA. Fertilization, hatching rate were analyzed using one-way ANOVA after a square root of arcsine transformation. LSD’s multiple comparisons were carried out when significant difference was found in the one-way ANOVA. One-way Kruskal–Wallis ANOVA was used to analyze larval width, stomach width and post-oral arm length/body rod length, because of the non-normal distribution and/or heterogeneity of variance of the data. Pairwise multiple comparisons were followed using Dunn–Bonferroni post hoc method when significant difference was found in the one-way Kruskal–Wallis ANOVA. All data are expressed as mean values ± standard deviation (mean ± SD). All analyses were done with SPSS 21.0 statistical software. A probability level of *P* < 0.05 was considered statistically significant.

## Results

### Egg diameter

**Figure 2:**
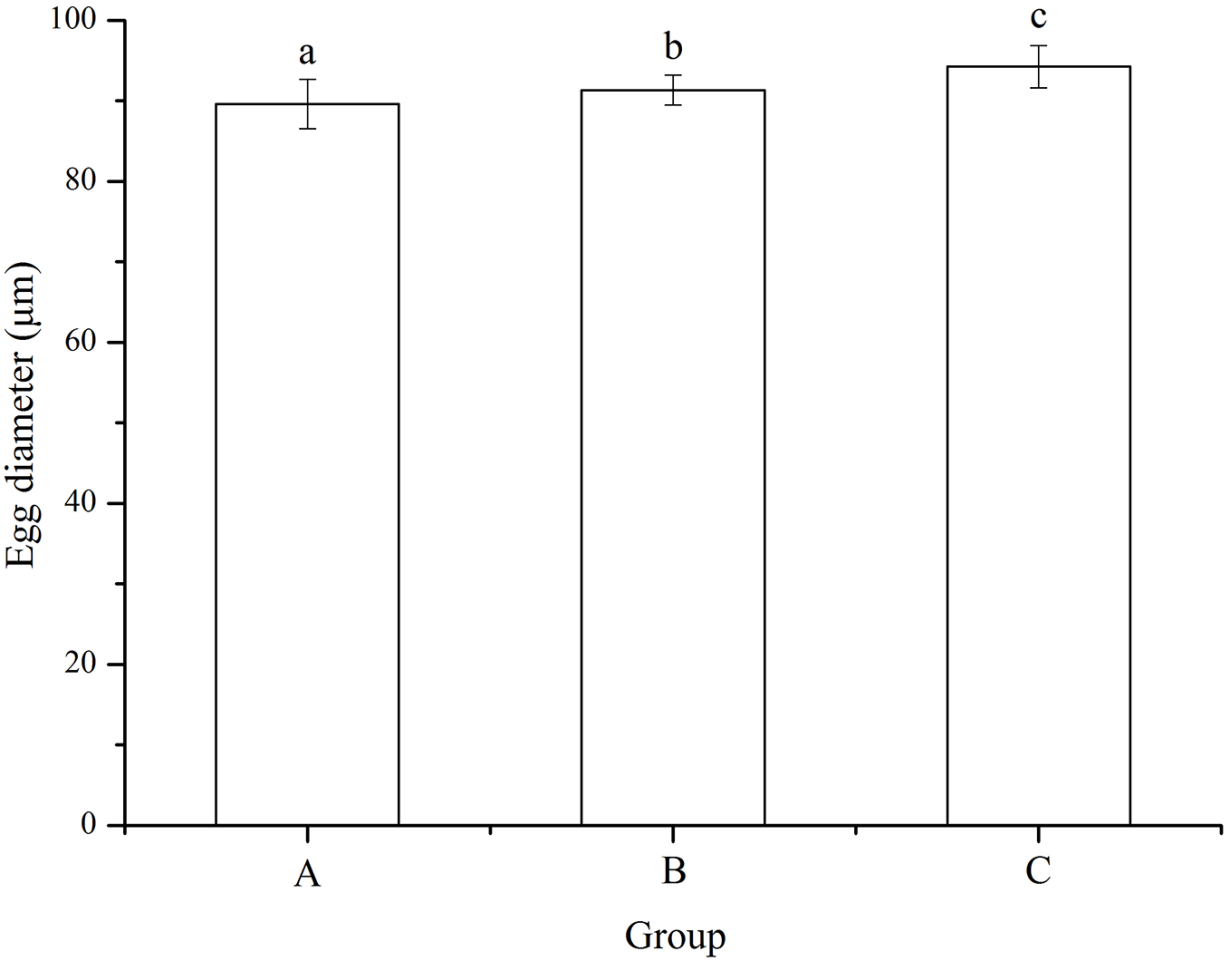
Egg diameter of *Strongylocentrotus intermedius* used for mating groups A (♀0 × ♂0 μW · cm^−2^), B (♀20 × ♂20 μW · cm^−2^) and C (♀20 × ♂0 μW · cm^−2^). Different letters above the bars represent significant difference (*P* < 0.05) among experimental groups.

No significant difference was found among families (*P* = 0.055). Eggs used for mating group C (94.24 ± 2.63 μm) was significantly larger than those for mating designs A (89.61 ± 3.05 μm) and B (91.34 ± 1.88 μm) (*P* < 0.001, *P* < 0.001, Fig. 2). In addition, egg diameter of group B was significantly larger than that of group A (*P* = 0.008, Fig. 2).

### Fertilization and hatching rates

**Figure 3:**
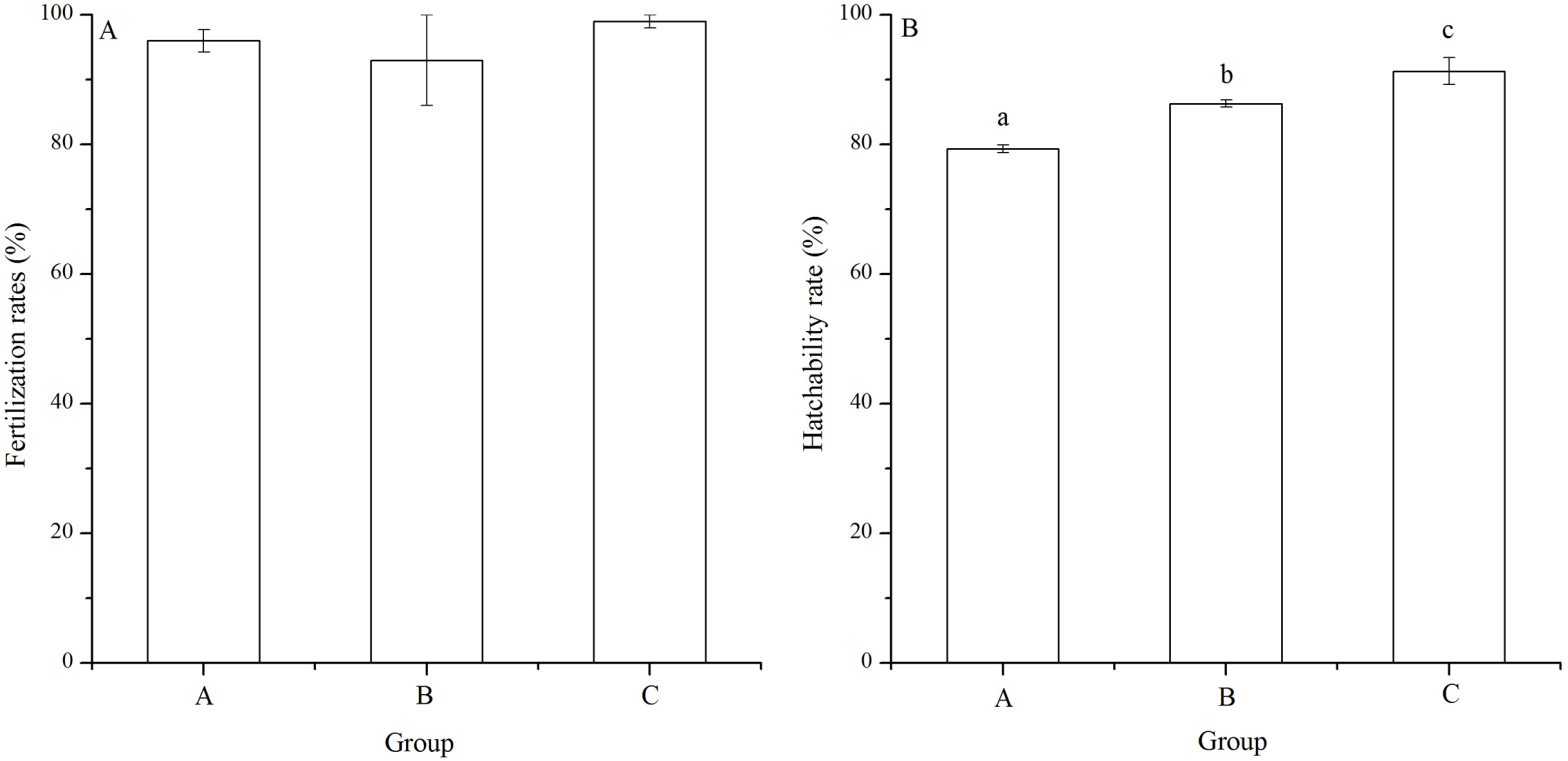
Fertilization rates (A) and hatching rates (B) of *Strongylocentrotus intermedius* in mating groups A (♀0 × ♂0 μW · cm^−2^), B (♀20 × ♂20 μW · cm^−2^) and C (♀20 × ♂0 μW · cm^−2^). No significant difference *(P* < 0.05) was found among experimental groups in (A). Different letters above the bars represent significant difference (*P* < 0.05) among experimental groups in (B).

There was no significant difference of fertilization rates among the mating groups (*P* = 0.112, Fig. 3A). On average, fertilization rates were over 90% in all groups (Fig. 3A).

The hatching rate of group C (91.33% ± 2.08%) was significantly higher than those of groups A (79.33% ± 0.58%) and B (86.33% ± 0.58%) (*P* < 0.001, *P* = 0.004). In addition, hatching rate of group B was significantly higher than that of group A (*P* = 0.002, Fig. 3B).

### Larval length and width

**Figure 4:**
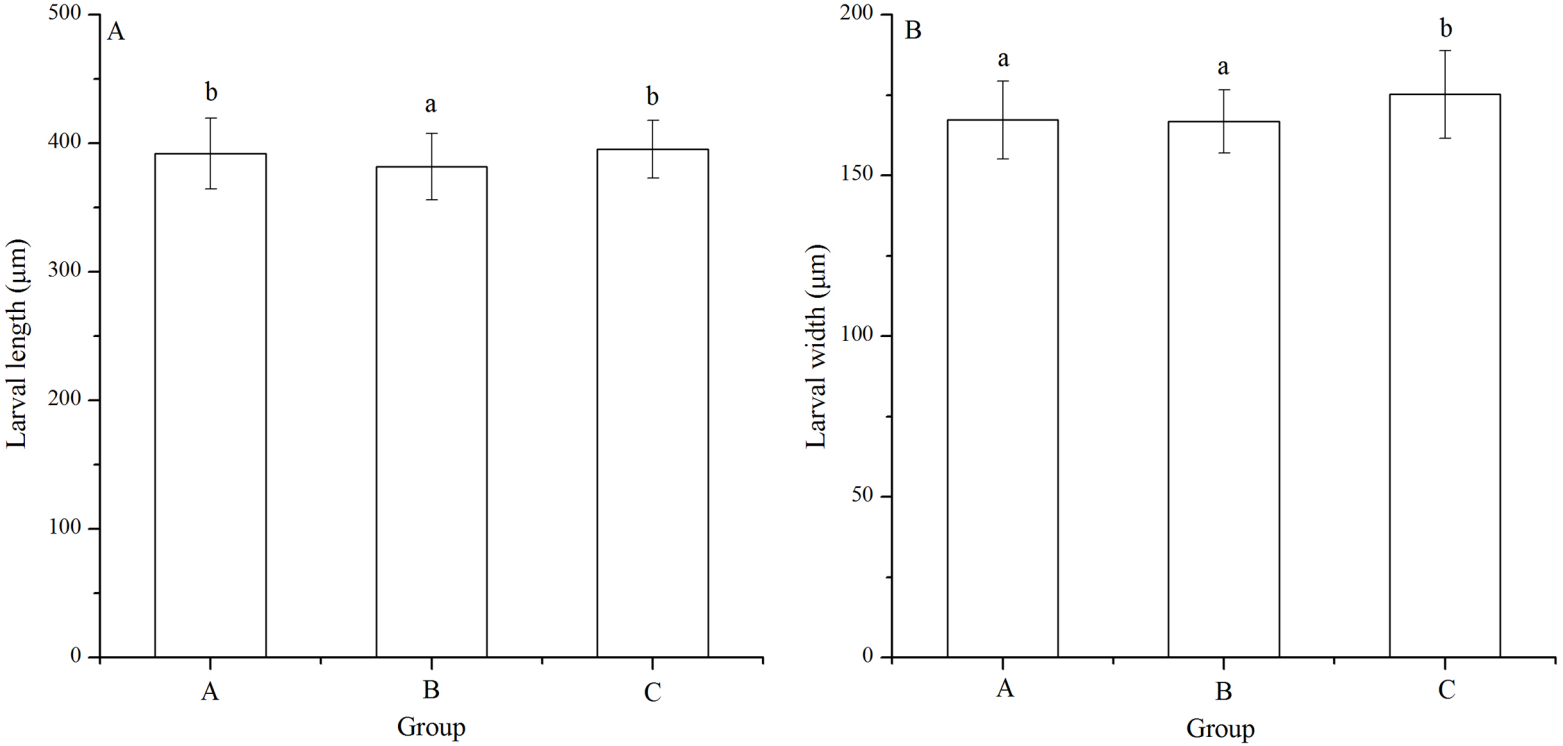
Larval length (A) and width (B) of *Strongylocentrotus intermedius* in mating groups A (♀0 × ♂0 μW · cm^−2^), B (♀20 × ♂20 μW · cm^−2^) and C (♀20 × ♂0 μW · cm^−2^). Different letters above the bars represent significant difference (*P* < 0.05) among experimental groups.

Significant difference in larval length was found among families (*P* < 0.001). The *Strongylocentrotus intermedius* of group B (381.87 ± 25.94 μm) showed significantly shorter larval length than groups A (392.06 ± 27.70 μm) and C (395.42 ± 22.39 μm) (*P* < 0.001, *P* < 0.001, Fig. 4A). However, there was no significant difference of larval length between groups A and C (*P* = 0.229, Fig. 4A)

Larval width of group C (175.22 ± 13.61 μm) was significantly greater than those of groups A (167.29 ± 12.11 μm) and B (166.80 ± 9.84 μm) (*P* < 0.001, *P* < 0.001, Fig. 4B). However, there was no significant difference of larval width between groups A and B (*P* = 1.000, Fig. 4B).

### Stomach length and width

**Figure 5:**
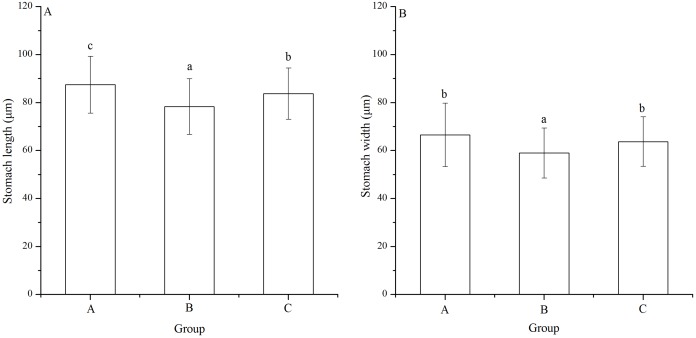
Stomach length (A) and width (B) of *Strongylocentrotus intermedius* larvae in mating groups A (♀0 × ♂0 μW · cm^−2^), B (♀20 × ♂20 μW · cm^−2^) and C (♀20 × ♂0 μW · cm^−2^). Different letters above the bars represent significant difference (*P* < 0.05) among experimental groups.

Significant difference was found in stomach width among families (*P* < 0.001). Stomach length of group B (78.33 ± 11.55 μm) was significantly shortest among the groups (*P* < 0.001, *P* < 0.001, Fig. 5A). In addition, group A (87.47 ± 11.86 μm) showed significantly larger stomach length than group C (83.76 ± 10.67 μm) (*P* = 0.005, Fig. 5A).

Group B (58.97 ± 10.45 μm) also had significantly shortest stomach width among the groups (*P* < 0.001, *P* < 0.001, Fig. 5B). However, there was no significant difference of stomach width between groups A (66.52 ± 13.21 μm) and C (63.71 ± 10.31 μm) (*P* = 0.268, Fig. 5B).

### Post-oral arm length, body rod length and post-oral arm length/body rod length

**Figure 6:**
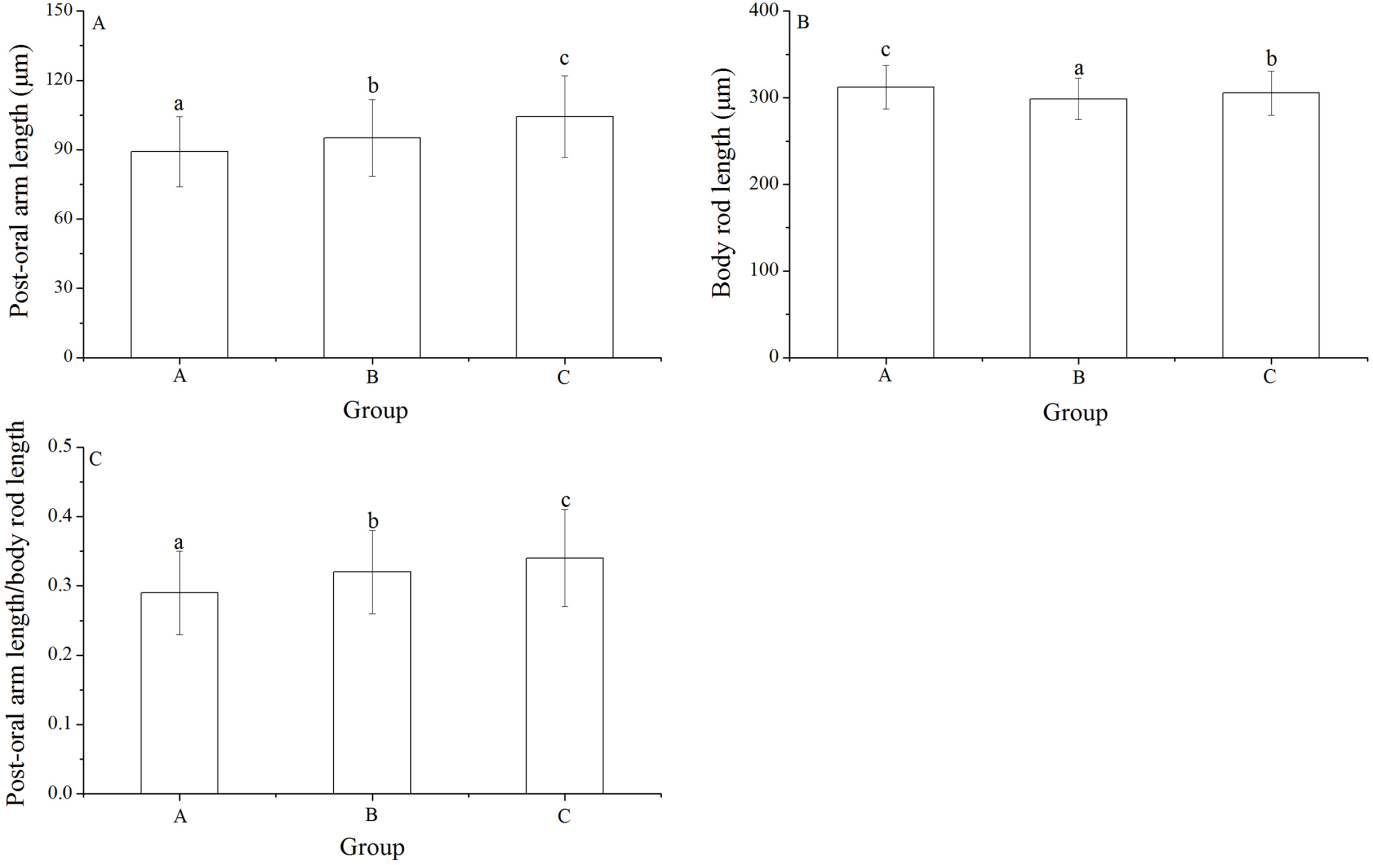
Body rod length (A), post-oral arm length (B) and body rod length/post-oral arm length (C) of *Strongylocentrotus intermedius* larvae among mating groups. Different letters above the bars represent significant difference (*P* < 0.05) among experimental groups.

Significant differences was found in post-oral arm length and body rod length among families (*P* < 0.001, *P* = 0.003). Post-oral arm length was significantly shortest in group A (89.13 ± 15.23 μm) than in groups B (95.16 ± 16.61 μm) and C (104.35 ± 17.71 μm) (*P* = 0.001, *P* < 0.001, Fig. 6A). Body rod length of group A (312.19 ± 25.19 μm), however, was significantly greater than those of groups B (298.61 ± 23.66 μm) and C (305.31 ± 25.31 μm) (*P* < 0.001, *P* = 0.015, Fig. 6B). Consistently, group A (0.29 ± 0.06) showed significantly lowest post-oral arm length/body rod length among the experimental groups (group B: 0.32 ± 0.06, group C: 0.34 ± 0.07) (*P* = 0.001, *P* < 0.001, Fig. 6C).

## Discussion

Ultraviolet-B radiation is an increasing threat to marine invertebrates in shallow waters (Day & Neale, 2002; Manney et al., 2011). However, current information is limited in the real-time effects on behaviors (Sigg, Lloyd-Knight & Boal, 2007), cellular responses (Lu & Wu, 2005), DNA damage (Mitchell, Adams-Deutsch & Olson, 2009), reproduction (Kane & Pomory, 2001) and development (Bonaventura et al., 2006) of marine organisms. Transgenerational effects are therefore essential for the full understanding of phenotypic plasticity and evolutionary adaptation of marine invertebrates in the changing ocean (Ross, Parker & Byrne, 2016). For the first time, we reported both positive and negative transgenerational effects of UV-B radiation on egg size, fertilization, hatchability and larval size in sea urchins.

In the present study, sea urchins exposed to short-term (1 h) UV-B radiation (20 μW · cm^−2^) spawned significantly larger eggs than those not exposed. Lamare et al. (2006) reported that the effects of UV-B radiation on different populations of sea urchins vary with latitudes both within and between species. Egg size was significantly different between geographic populations of the *Echinometra* sea urchins *(*McAlister & Moran, 2012). Thus, UV-B radiation is probably involved in the population difference of egg size, although it was not measured at the collection sites (McAlister & Moran, 2012). The sea urchin *Heliocidaris tuberculata* spawns small eggs and has feeding larvae, while *H. erythrogramma* spawns large eggs and has non-feeding larvae (Byrne et al., 1999; Foo, Deaker & Byrne, 2018), suggesting that larger eggs are more suitable for the consequent limited food conditions (Emlet & Hoegh-Guldberg, 1997). In the present study, the larger eggs are consistent with significantly less food consumption of *Strongylocentrotus intermedius* exposed to UV-B radiation (Zhao et al., 2018a). We hypothesize that transgenerational adaptation and/or acclimation occurs on increasing egg size to adapt the condition of limited food consumption. Further studies are definitely essential to test this hypothesis.

Ultraviolet-B radiation did not transgenerationally affect the fertilization of *Strongylocentrotus intermedius*. This is consistent with our previous finding that long-term elevated temperature showed no transgenerational effect on fertilization of *Strongylocentrotus intermedius* (Zhao et al., 2018b). Together with the previous study, the present result indicates the robustness of fertilization, in despite of their parents are exposed to adverse environments.

Ultraviolet-B radiation significantly reduced the hatchability of the copepod *Paracyclopina nana* (Won et al., 2014). However, significantly higher hatching rates were found in the groups exposed to UV-B radiation. The increased hatching rate probably resulted from the relatively short radiation duration (1 h) and long-term recovery (10 months) in the present study. The current result suggests a positive transgenerational effect of UV-B radiation on the fitness of sea urchins. The trend of hatching rates was well consistent with that of egg diameter. The larger eggs and the greater region of cells at the core that receives little or no UV are in accordance with the greater proportion of unexposed DNA and the lower CPD load per unit DNA (Browman et al., 2003) Besides, UV-B radiation probably keeps microbial infection of eggs at a low level (Skresleta et al., 2005; Hansen & Olafsen, 1989), thereby leading to improved embryo survival (Skresleta et al., 2005; Hansen & Olafsen, 1989). This probably promoted the consequently increasing hatchability of sea urchins. The increasing of egg size and consequent hatchability clearly indicates of the adaptation to UV-B radiation in sea urchins.

*Strongylocentrotus intermedius*, whose parents were both exposed to short-term UV-B radiation (20 μW ∙ cm^−2^), showed significantly shortest larval length and width. Although carotenoids in *Saccharina japonica* as a powerful antioxidant that helps animals to minimize oxidative damage (Matsuno & Tsushima, 2001), a negative transgenerational effect of UV-B radiation on larval size of *Strongylocentrotus intermedius* occurs. This enriches our previous findings that UV-B radiation at 20 μW ∙ cm^−2^ is dangerous to marine invertebrates (at least *Strongylocentrotus intermedius*) (Zhao et al., 2018a). Interestingly, larval length was not significantly reduced in the sea urchins of group C, whose dams were exposed to UV-B radiation while sires were not. Further, larval width was significantly largest in sea urchins of group C. Carroll & Shick (1996) noted that MAAs, the most important group of photoprotective pigments in larval stage of echinoderms, which is endowed to the eggs maternally. In the sea urchin *Sterechinus neumayeri*, the greatest MAAs concentrations were found in the ovaries (Karentz, Dunlap & Bosch, 1997; McClintock & Karentz, 1997). UV-induced negative effects in the offspring of sea urchins was clearly correlated with the absence of MAAs (Häder et al., 2007). Our results showed group B with the shortest body size while larval size of group C without adverse effects. This suggests that MAAs of dams is more robustness than that of sires under UV-B radiation (20 μW ∙ cm^−2^). Although dams can provide a normal amount of MAAs, the amount provided by the sires is too less. Thus, the offspring larvae don’t have sufficient concentrations of MAAs to maintain their normal individual development, resulting in smaller larval size in group B.

Stomach size is important for larval development and growth (Chang et al., 2004). Sea urchins whose both parents were exposed to short-term UV-B radiation (20 μW ∙ cm^−2^) showed significantly shortest stomach length and width. This result is well consistent with the reduced larval size in the present study, indicating a negative transgenerational effect of UV-B radiation on the stomach size. Stomach width was not significantly reduced in the sea urchins of group C, although stomach length was significantly impacted. This result confirms the conclusion that sires may play an essential role in transgenerational effects of UV-B radiation of sea urchins.

Phenotypic plasticity has important ecological and evolutionary implications on early developmental stages of marine invertebrates to address temporal changes in abiotic environmental conditions (McAlister & Miner, 2018). In this sense, food limitation induces longer post-oral arm length, both absolutely and relative to body rod length (Adams et al., 2011; McAlister & Miner, 2018). Because in order to avoid UV-B radiation, covering and shading behaviors of sea urchins may reduce movement and hence the ability of feeding (Kehas, Theoharides & Gilbert, 2005; Dumont et al., 2007; Burnaford & Vasquez, 2008). In the present study, post-oral arm length/body rod length significantly increased in larvae whose parents were exposed to short-term UV-B radiation, indicating a positive transgenerational effect. This result is well consistent with the hypothesis that the larger arms are an adaptation matched to the less food consumption of their parents. Consistently, elevated temperature transgenerationally increased post-oral arm length/body rod length in *Strongylocentrotus intermedius*, whose parents also fed significantly less (Zhao et al., 2018a). Further, as to the proximate mechanisms causing the observed effects, Pahkala, Laurila & Merila (2001) pointed that a trade-offs between energy distributions among different functions in vivo could be involved in carry-over effects of UV-B radiation on larval fitness in *Rana temporaria*. Thus, we suspect a trade-off between the energy allocated to foraging behavior and that allocated to cellular UV-B damage repair could be involved. Thus, it’s possible that the increased post-oral arm length/body rod length helps *Strongylocentrotus intermedius* get food around with as little displacement as possible, helping them save energy for UV-B damage repair in vivo. In addition, significant family differences were found in larval length, stomach width, post-oral arm length and body rod length (with the exception of egg diameter). This is consistent with the finding that larval growth of the sea urchin *Strongylocentrotus intermedius* including larval size, stomach size, post-oral arm length and body rod length was significantly influenced by the degree of inbreeding of the families (Zhao et al., 2016).

In conclusion, *Strongylocentrotus intermedius* exposed to short-term (1 h) UV-B radiation (20 μW ∙ cm^−2^) showed positive transgenerational effects and adaptation on egg size, hatching rate and post-oral arm length of larvae. Negative transgenerational effects were found in body length, stomach length and stomach width of larvae whose parents were exposed to UV-B radiation. Sires probably play essential roles in transgenerational effects of UV-B radiation. The present study provides valuable information into transgenerational effects of UV-B radiation on fitness related traits of sea urchins (at least *Strongylocentrotus intermedius*).

## Supplemental Information

10.7717/peerj.7598/supp-1 Supplemental Information 1 Egg size.

10.7717/peerj.7598/supp-2 Supplemental Information 2 Fertilization and hatchability.

10.7717/peerj.7598/supp-3 Supplemental Information 3 Gonad developmental stage.

10.7717/peerj.7598/supp-4 Supplemental Information 4 Larval size.

## Additional Information and Declarations

### Data Availability

The following information was supplied regarding data availability: The raw data is available in the Supplemental Files.
